# Sulphate in Pregnancy

**DOI:** 10.3390/nu7031594

**Published:** 2015-03-04

**Authors:** Paul A. Dawson, Aoife Elliott, Francis G. Bowling

**Affiliations:** 1 Mater Research Institute, Level 4, Translational Research Institute, University of Queensland, 37 Kent St, TRI, Woolloongabba, QLD 4102, Australia; E-Mails: aelliott@mmri.mater.org.au (A.E.); francis.bowling@dhhs.tas.gov.au (F.G.B.); 2 Mater Children’s Hospital, Mater Health Services, South Brisbane, QLD 4101, Australia

**Keywords:** sulphate, sulphonation, foetal development, pregnancy, gestation

## Abstract

Sulphate is an obligate nutrient for healthy growth and development. Sulphate conjugation (sulphonation) of proteoglycans maintains the structure and function of tissues. Sulphonation also regulates the bioactivity of steroids, thyroid hormone, bile acids, catecholamines and cholecystokinin, and detoxifies certain xenobiotics and pharmacological drugs. In adults and children, sulphate is obtained from the diet and from the intracellular metabolism of sulphur-containing amino acids. Dietary sulphate intake can vary greatly and is dependent on the type of food consumed and source of drinking water. Once ingested, sulphate is absorbed into circulation where its level is maintained at approximately 300 μmol/L, making sulphate the fourth most abundant anion in plasma. In pregnant women, circulating sulphate concentrations increase by twofold with levels peaking in late gestation. This increased sulphataemia, which is mediated by up-regulation of sulphate reabsorption in the maternal kidneys, provides a reservoir of sulphate to meet the gestational needs of the developing foetus. The foetus has negligible capacity to generate sulphate and thereby, is completely reliant on sulphate supply from the maternal circulation. Maternal hyposulphataemia leads to foetal sulphate deficiency and late gestational foetal death in mice. In humans, reduced sulphonation capacity has been linked to skeletal dysplasias, ranging from the mildest form, multiple epiphyseal dysplasia, to achondrogenesis Type IB, which results in severe skeletal underdevelopment and death *in utero* or shortly after birth. Despite being essential for numerous cellular and metabolic functions, the nutrient sulphate is largely unappreciated in clinical settings. This article will review the physiological roles and regulation of sulphate during pregnancy, with a particular focus on animal models of disturbed sulphate homeostasis and links to human pathophysiology.

## 1. Introduction

**Figure 1:**
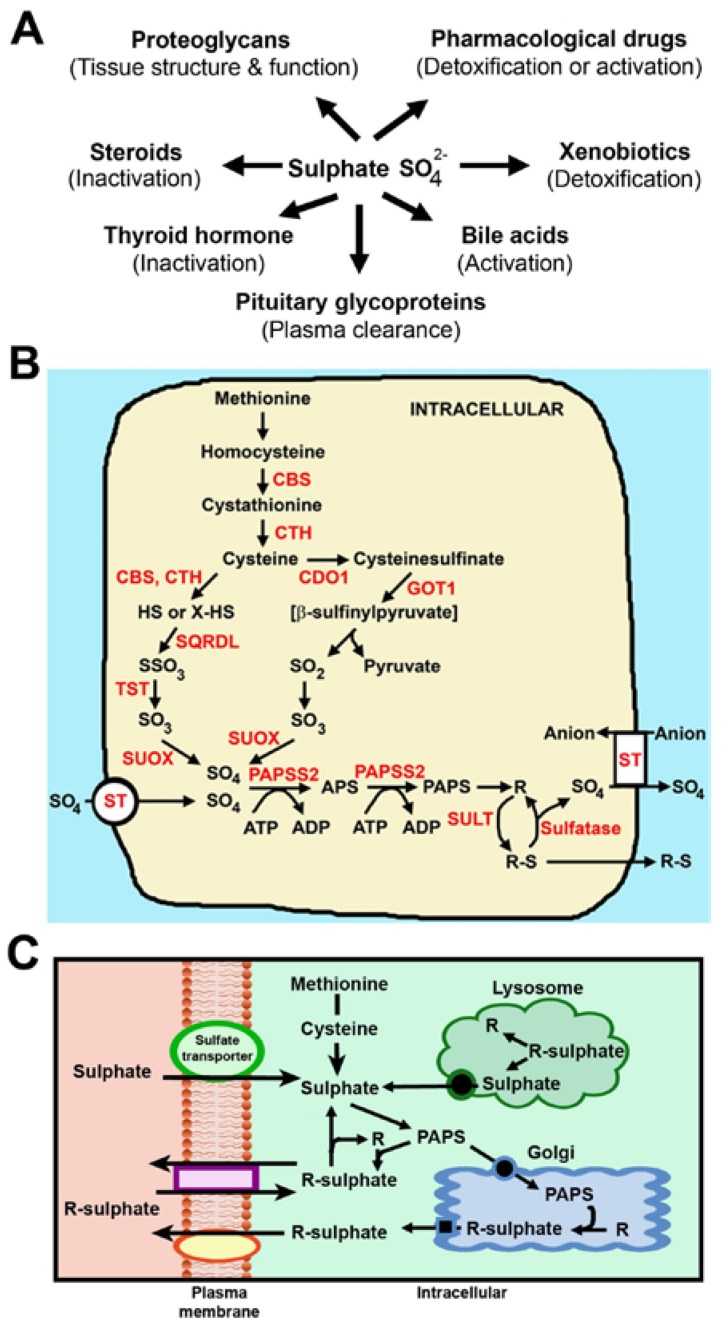
Biological roles of sulphate and pathways of sulphate homeostasis. (**A**) Sulphonation contributes to numerous cellular and metabolic functions in human physiology; (**B**) Pathways of intracellular sulphate generation and sulphonation. Methionine is converted to cysteine via the transsulphuration pathway involving cystathionine β-synthase (CBS) and cystathionine γ-lyase (CTH). Cysteine is converted to sulphate via two pathways: A minor pathway involving CBS, CTH, sulphide quinone reductase-like (SQRDL), thiosulphate sulphurtransferase (TST) and sulphite oxidase (SUOX); and a major pathway involving cysteine dioxygenase (CDO), glutamic-oxaloacetic transaminase 1 (GOT1) and SUOX. ST, Sulphate transporters; PAPSS2, PAPS synthetase; SULT, sulphotransferases; R represents those substrates shown in (A); (**C**) Flux of intracellular sulphate and sulphonated molecules. In adults and children, sulphate is obtained from: (i) extracellular sources via sulphate transporters; (ii) catabolism of methionine and cysteine; (iii) hydrolysis of proteoglycans in the lysosome; and (iv) sulphatase-mediated removal of sulphate from substrates in the cytosol.

Sulphate is an obligate nutrient for numerous metabolic and cellular processes, particularly in foetal growth and development [1]. The conjugation of sulphate (sulphonation) to certain endogenous molecules, including steroids (e.g., oestrogens) and thyroid hormone leads to their inactivation [2,3,4]. Importantly, the ratio of sulphonated (inactive) to unconjugated (active) hormones plays a role in modulating endocrine function, and therefore foetal and maternal physiology during pregnancy [3]. Additionally, sulphonation of structural components such as chondroitin sulphate, heparan sulphate and cerebroside sulphate is essential for the development and maintenance of tissue structure and function [5,6]. Furthermore, the foetal liver expresses abundant levels of sulphotransferases that mediate the sulphonation and clearance of xenobiotics and certain pharmacological drugs that are potentially detrimental to foetal development [7,8]. This latter role for sulphate is particularly important in human and animal gestation, as the developing foetus has negligible capacity to detoxify xenobiotics via the glucuronidation pathway that is largely inactive in the prenatal period [9,10]. Over the past few decades, numerous roles for sulphate have been described in human physiology (Figure 1A) [11]. However, despite these important physiological roles, sulphate is not routinely measured in clinical settings. Accordingly, this review highlights our current knowledge on sulphate nutrition with a particular focus on the roles and regulation of sulphate in human and animal gestation.

## 2. Sulphate is Obtained from the Diet

Sulphonation relies on a sufficient supply of sulphate, which is obtained from the diet as free inorganic sulphate (SO_4_^2−^) or generated from sulphonated compounds and the sulphur-containing amino acids, methionine and cysteine [5,12]. A well-balanced diet contributes approximately one third of estimated average body sulphate requirements (0.2–1.5 g SO_4_^2−^/day) [13,14,15,16]. Certain foods, including brassica vegetables and commercial breads contain a high sulphate content (>0.9 mg/g), whereas low sulphate levels (<0.1 mg/g) are found in some foods such as fresh apples and oranges [15]. In addition, the sulphate content of drinking water can vary greatly, from negligible levels in demineralised bottle water to >500 mg/L in water from spring-fed wells and dams [13,14,15]. Sulphate levels exceeding 500 mg/L of drinking water can result in an unpleasant taste, although some individuals are more sensitive to lower concentrations [16]. Inhalation of sulphate in air is estimated to contribute trace amounts (0.01–0.04 mg SO_4_^2−^/day) for adults [17]. In addition, certain prenatal multivitamin-multimineral supplements contain sulphate, primarily in the form of cupric sulphate anhydrous, zinc sulphate and manganese sulphate, with approximately 25–40 mg SO_4_^2−^/tablet.

Sulphate is one of the least toxic anions, with reported lethal doses being 45 g potassium sulphate or zinc sulphate for humans, and a minimal lethal dose of 200 mg/kg magnesium sulphate in mammals [18]. Osmotic diarrhoea has been reported in healthy adult males when they consumed 8 g of sodium sulphate (6.7 g sulphate) as a single dose, and in infants consuming sulphate concentrations >600 mg/L of water with an estimated sulphate intake of ≈66 mg/kg/day [19,20]. In addition, a self-reported laxative effect was reported in most adults consuming water with levels of sulphate 1000 to 2000 mg/L (approximately 14 to 29 mg/kg body weight) [16]. Similar findings of sulphate-induced osmotic diarrhoea have been reported in animal studies [21]. High concentrations of ingested magnesium sulphate have also been linked to osmotic diarrhoea but this is most likely due to the poor absorption of magnesium, as sulphate absorption is much higher [22,23]. Magnesium sulphate is also used for seizure prevention in preeclampsia or eclampsia, as well as a tocolytic agent, being administered i.v. to women shortly before preterm birth [24]. However, this treatment is rather unpleasant for some women with approximately 8% of women requiring cessation of treatment due to intolerable side-effects, including nausea, vomiting, flushing sweating and palpitations [24]. Oral supplements of ferrous sulphate (100 mg FeSO_4_ per capsule per day, ≈63 mg sulphate per capsule) are prescribed to treat iron deficiency anaemia in pregnancy. However, ferrous sulphate can be irritating to the gastrointestinal tract [25], which is largely attributed to the ferrous ions [26]. Comparative data on the effects of different iron preparations have shown that ferrous sulphate may elicit stronger inflammatory processes in the pregnant rat and foetus, when compared to ferrous fumarate [27]. These findings warrant further investigations of ferrous sulphate and other iron preparations in human pregnancy. Whilst the above findings suggest that caution may be warranted in consuming sulphate levels significantly above that found in most foods, there are currently insufficient data to identify an upper intake level to cause adverse effects to human health. Nonetheless, both food (≈0.85 g SO_4_^2−^/day) and drinking water (≈0.78 g SO_4_^2−^/day) provide an important source of sulphate [16], particularly in late gestation when foetal sulphate demands are increasing.

The nutritional value of sulphate in bolstering the growth of laboratory rodents was first reported almost a century ago [28]. More recent animal studies have shown that restricting sulphate in both food and water can lead to sulphate deficiency and reduced growth, which can be reversed by sulphate supplementation [29,30,31,32]. In addition, high dietary sulphate intake and administration of sulphate salts (MgSO_4_, Na_2_SO_4_ and ZnSO_4_) can lead to increased circulating sulphataemia and enhanced sulphonation capacity [33,34,35,36,37,38,39]. However, there is currently no recommended dietary intake for inorganic sulphate in humans, mainly because sulphate can be generated from the sulphur-containing amino acids.

## 3. Generation of Sulphate from Intracellular Metabolism

Protein is comprised of approximately 4% of the sulphur-containing amino acids methionine and cysteine [40]. Considering that the recommended daily intake of protein for 19–50 year olds in pregnancy is 0.8–1.0 g/kg [41], then the estimated amount of sulphate generated from protein is approximately 1.7 g/day. Both adults and children have the capacity to metabolise methionine and cysteine to sulphate [1]. Methionine is converted to cysteine via the transsulphuration pathway, and cysteine is further oxidised to sulphate via 2 pathways: A minor pathway of sulphate generation via cystathionine γ-lyase (CTH) and cystathionine β-synthase (CBS); and a major pathway via cysteine dioxygenase (CDO) (Figure 1B) [42]. Earlier studies reported the absence of CTH and CDO in human and rodent foetal liver, indicating that the developing foetus has a limited capacity to generate sulphate from the sulphur-containing amino acids [43,44]. This raises the question of which sources supply the high foetal demands for sulphate during pregnancy?

**Figure 2:**
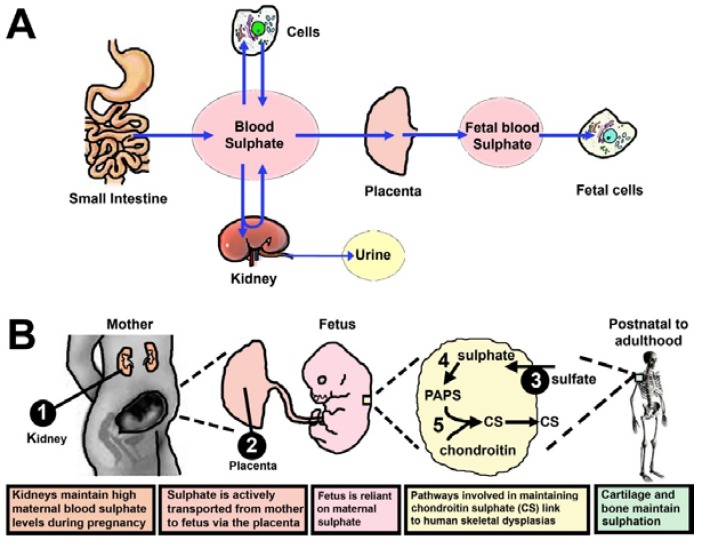
Fluxes of sulphate between tissues. (**A**) Contribution of the small intestine, kidneys and cells to sulphate homeostasis (**B**) Maternal, foetal and postnatal contributions to chondroitin sulphation. Disruption of pathways that maintain a sufficient supply of sulphate for chondrocytes (steps 1–3) or intracellular sulphonation of chondroitin (steps 4–5) lead to chondrodysplasias.

In adults and children, circulating sulphate levels are influenced by absorption in the small intestine, reabsorption in the kidneys, and uptake into cells throughout the body (Figure 2A) [1]. Circulating sulphate is a major source of sulphate for supplying the intracellular sulphonation of substrates in the cytoplasm (steroids, hormones, xenobiotics and proteins) or golgi apparatus (proteoglycans) [45,46]. However, the overall flux of intracellular sulphate is maintained by four pathways (Figure 1C): (i) Extracellular sulphate from circulation is transported through the plasma membrane of cells via sulphate transporters; (ii) Methionine and cysteine are catabolised to sulphate; (iii) Sulphate is removed from proteoglycans via sulphatase enzymes in the lysosome and then transported into the cytoplasm; and (iv) Cytosolic sulphatases remove sulphate from sulphonated molecules. The latter three sources have negligible or low contributions to the foetal intracellular sulphate pool, which is therefore reliant on extracellular sources sulphate [1]. In addition, the developing foetus has immature renal reabsorption and intestinal absorption capacities, highlighting the obligate requirements for supplying sulphate from mother to foetus via the placenta throughout gestation.

## 4. Sulphate Is Supplied from Mother to Foetus

During human and rodent pregnancy, maternal circulating sulphate levels increase by more than twofold to meet the gestational needs of the growing foetus [47,48], and this is remarkable because most plasma ion concentrations usually decrease slightly in pregnancy due to haemodilution [49] and speaks to its crucial role in foetal development. The increased maternal blood sulphate levels arise from increased sulphate reabsorption in the mother’s kidneys (Figure 2B) [47,48], which is mediated by increased renal expression of the *SLC13A1* gene (aka NaS1, sodium sulphate transporter 1) [50]. Disruption of *SLC13A1* in humans and mice causes sulphate wasting into the urine [51,52], and this greatly reduces blood sulphate levels (hyposulphataemia). In mice, loss of the *Slc13a1* gene leads to behavioural abnormalities (reduced memory and olfactory function, and increased anxiety), reduced brain serotonin levels, growth retardation, impaired gastrointestinal mucin sulphonation and enhanced acetaminophen-induced liver toxicity [33,51,52,53,54,55,56,57,58]. In addition, pregnant female *Slc13a1* null mice exhibit hyposulphataemia throughout gestation, which leads to foetal sulphate deficiency and mid-gestational miscarriage [48].

A related gene *SLC13A4* (aka NaS2, sodium sulphate transporter 2) was recently found to be the most abundant sulphate transporter in the human and mouse placenta [50,59]. *SLC13A4* is localised to the syncytiotrophoblast layer of the placenta, the site of maternal-foetal nutrient exchange, where it is proposed to be supplying sulphate from mother to foetus [59]. Loss of placental SLC13A4 in mice leads to severe foetal developmental abnormalities and late gestational foetal death, highlighting the obligate requirement of sulphate for healthy foetal growth and development [60].

Over the past decade, interest in the roles and regulation of sulphate during pregnancy has expanded following the characterisation of growth restriction and foetal demise in animal models of reduced sulphonation capacity [11]. For example, mice lacking the Sult1e1 oestrogen sulphotransferase exhibit mid-gestational foetal loss [61]. Sult1e1 is expressed in the placenta where it is essential for generating the sulphonated forms of estrone sulphate, estradiol-3-sulphate and estriol sulphate. Foetal loss and impaired foetal growth have also been linked to several other sulphotransferases and sulphatases that maintain the required biological ratio of sulphonated to unconjugated proteins and proteoglycans [11]. Despite the evidence from animal studies that show the physiological importance for sulphate during pregnancy, there are no routine measurements of sulphate in clinical settings.

In humans, free inorganic sulphate (SO_4_^2−^) is the fourth most abundant anion in circulation (approximately 300 μmol/L) [62]. Early studies reported a twofold increase in plasma sulphate levels in pregnant women [35,63,64,65]. More recent studies used a validated ion chromatography method to establish reference ranges for maternal plasma sulphate levels in early (10–20 weeks) and late (30–37 weeks) gestation, as well as cord plasma sulphate levels from healthy term pregnancies [47]. These data will now enable clinical investigations into the outcomes of low plasma sulphate levels in mother and child, and will most likely expand our current knowledge into the consequences of sulphate deficiency, particularly skeletal development, which is sensitive to sulphate deficiency.

## 5. Reduced Sulphonation Capacity Perturbs Skeletal Growth and Development

In mammals, sulphonated proteoglycans are an essential component of extracellular matrices throughout the body, particularly in connective tissues [66,67]. The sulphate content of proteoglycans influences cell signalling function and the structural integrity of tissues [5]. Highly sulphonated glycoproteins, including chondroitin proteoglycan (CSPG), play important roles in the developing skeleton, with links to modulation of the Indian Hedgehog signalling pathway [68]. Importantly, sulphonation of CSPGs in chondrocytes is essential for normal skeletal growth and development, and several skeletal disorders have been attributed to genetic defects that lead to decreased sulphonation capacity [11].

Chondrocytes rely on an abundant supply of extracellular sulphate, to meet the intracellular demands for CSPG sulphonation (Figure 2B). Sulphate is transported into chondrocytes via the SLC26A2 sulphate transporter (step 3 of Figure 2B) [69]. More than 30 mutations in the human *SLC26A2* gene have been linked to chondrodysplasias [70], with the underlying metabolic defect being reduced sulphonation of chondroitin in chondrocytes [71]. Mutant *Slc26a2* mice also exhibit chondrodysplasias which mimics the biochemical and morphological phenotypes found in humans [71,72,73]. Treatment of the mutant *Slc26a2* mice with dietary *N*-acetyl cysteine, showed increased proteoglycan sulphonation and improved skeletal phenotypes [31], suggesting that thiol-containing compounds can bolster the intracellular sulphate levels needed for sulphonation of CSPGs.

Loss of PAPS (3′-phosphoadenosine 5′-phosphosulphate) synthetase has also been linked to impaired CSPG sulphonation and skeletal dysplasias [74]. PAPS is the universal sulphonate donor for all sulphonation reactions and its formation relies on a sufficient intracellular supply of sulphate (step 4 in Figure 2B) [75]. Mammalian genomes contain two PAPS synthetase genes, *PAPSS1* and *PAPSS2* [76,77,78]. *PAPSS2* has been linked to human pathophysiology, with similar skeletal phenotypes found in *Papss2* mutant mice [76,78]. In addition, disruption of the zebrafish PAPS transporter gene (*PAPST1*, aka *pinscher*) leads to cartilage defects [79]. Skeletal phenotypes are also found in patients with mutations in the chondroitin 6-*O*-sulphotransferase gene (step 5 in Figure 2B) [80], showing that chondroitin sulphonation is important for maintaining healthy skeletal development. These findings highlight the importance of pathways that lead to chondroitin sulphation for healthy development, growth and maintenance of the skeleton.

Currently, there is no cure for the most severe skeletal dysplasia forms, atelosteogenesis Type II and achondrogenesis Type IB, which result in skeletal underdevelopment and death *in utero* or in the neonatal period [70]. The mild (multiple epiphyseal) and moderate (diastrophic dysplasia) forms of the disease are treated with orthopaedic and pain management but these patients face a lifetime of disability. Other genes including *PAPSS2* have involvement with abnormal skeletal growth and development in humans [76], and the clinical spectrum associated with *PAPSS2* and *SLC26A2* has further expanded to include knee osteoarthritis [81], suggesting that sulphation disorders are likely to be more prevalent than the estimated 2% of all skeletal dysplasias which is based on live births [82]. This is also relevant to recent studies that have linked the renal *Slc13a1* sulphate transporter gene, which is important for maintaining circulating sulphate levels, to skeletal dysplasias in animals [83,84]. These findings are likely to be relevant for human skeletal growth and development. Collectively, the lack of curative treatments for the skeletal sulphonation disorders leads to significant burden on families and community [85].

The biochemical basis of under-sulphation in the skeletal sulphation disorders is well established [69,70,86] and warrants approaches to the development of therapies for increasing sulphation capacity. Prenatal diagnosis of babies with nonlethal sulphation disorders is helpful for clinical geneticists, neonatologists, obstetricians and anaesthesiologists to plan delivery and improve postnatal outcomes. However, many of these surviving babies face life-long physical impairments, placing a huge burden on affected families [85]. Currently, there is no cure for individuals with skeletal sulphonation disorders. Conventional treatments, including orthopaedic intervention and pain management for the non-lethal forms are inadequate and warrant develop of new therapeutic approaches. In humans, there is a dosage effect of sulphonation capacity on clinical outcomes, with negligible/low sulphonation leading to the lethal and severe skeletal dysplasias, whereas moderate reductions in sulphation give rise to milder clinical outcomes [70]. The dosage effect suggests that strategies which can increase sulphonation capacity in the skeleton should ameliorate the clinical presentations. This is relevant to the high foetal demands for sulphate in mid- to late-gestation [1], which provides a window in gestation when sulphate supplementation therapies may potentially provide the most benefit for foetuses affected by a skeletal sulphation disorder. If simple low cost maternal dietary interventions, using sulphonated compounds, could increase sulphonation capacity in the developing foetal skeleton, then this could potentially have enormous benefits for ameliorating the skeletal phenotypes in affected individuals.

## 6. Conclusion

Sulphate is an obligate nutrient for healthy growth and development. Despite being essential for numerous cellular and metabolic processes in foetal development, its importance is largely underappreciated in clinical settings. Animal models have shown the devastating physiological outcomes of reduced sulphonation capacity on foetal growth and development, which is relevant to the established link with human chondrodysplasias. A sufficient supply of sulphate, either from the diet or from the sulphur-containing amino acids, needs to be supplied from mother to foetus, particularly in late gestation when foetal demands for sulphate are high. The development of a validated method for sulphate quantitation, together with recent data for maternal plasma sulphate reference ranges, now warrants further investigations into the consequences of nutrient sulphate deficiency in mother and child.
